# Increased risk of suicide after stroke: A population-based matched cohort study

**DOI:** 10.1177/17474930251379165

**Published:** 2025-09-05

**Authors:** Manav V Vyas, Claire de Oliveira, Gustavo Saposnik, Peter C Austin, Amy YX Yu, Olivia Haldenby, Jiming Fang, Corinne E Fischer, David Lipson, Fatima Quraishi, Moira K Kapral, Venkat Bhat

**Affiliations:** 1 Division of Neurology, Department of Medicine, University of Toronto, Toronto, ON, Canada; 2 St. Michael’s Hospital-Unity Health Toronto, Toronto, ON, Canada; 3 Institute of Health Policy, Management and Evaluation, Dalla Lana School of Public Health, University of Toronto, Toronto, ON, Canada; 4 ICES, Toronto, ON, Canada; 5 Campbell Family Mental Health Research Institute and Institute for Mental Health Policy Research, Centre for Addiction and Mental Health, Toronto, ON, Canada; 6 Department of Medicine (Neurology), University of Toronto, Sunnybrook Health Sciences Centre, Toronto, ON, Canada; 7 Department of Psychiatry, University of Toronto, Toronto, ON, Canada; 8 Department of Physical Medicine and Rehabilitation, Providence Health, University of Toronto, Toronto, ON, Canada; 9 Division of General Internal Medicine, Department of Medicine, University of Toronto, Toronto, ON, Canada; 10 Interventional Psychiatry Program, St. Michael’s Hospital-Unity Health Toronto, Toronto, ON, Canada

**Keywords:** Suicide, major depression, effect modification, hazards models, matched cohort study, retrospective, self-harm, death by suicide

## Abstract

**Background and Objectives:**

We examined the timing of suicide after stroke, the sociodemographic factors associated with the risk of suicide, and whether major depression modified the stroke–suicide association.

**Methods:**

We conducted a population-based retrospective cohort study of all adults in Ontario hospitalized for stroke between January 1, 2008, and December 31, 2017, who were matched 1:1 to controls from the general Ontario population on age, sex, neighborhood-level income, rurality, and comorbidities. Suicide, a composite of deliberate self-harm or death by suicide, was ascertained based on hospitalizations and emergency department visits. Cause-specific hazard models were used to evaluate the association between stroke and suicide, and major depression was treated as a time-varying covariate. Cause-specific hazard models evaluated the association between sociodemographic factors and suicide in stroke survivors. The modifying effect of major depression was assessed by adding an interaction term between stroke and major depression.

**Results:**

We included 64,719 matched pairs of patients with stroke and general population controls (45.4% female, mean age 71.4 years). In the 627,774 person-years follow-up, 436 cases and controls had an episode of self-harm or died by suicide, with 203 (67.4%) events in stroke survivors occurring after the first year. Compared to matched controls, stroke survivors had a higher rate of suicide (11.1 vs 3.2 per 10,000 person-years, hazard ratio (HR) 2.87; 2.35–3.51). The association between stroke and suicide did not vary by the presence of major depression (P_stroke*depression_ = 0.51). Suicide rates were elevated in younger stroke survivors (HR_18–40 vs_ _⩾_ _80 years_ 4.34; 2.48–7.61), those living in low-income neighborhoods (HR_lowest vs highest quintile_ 1.88; 1.30–2.70), and those with major depression (HR 12.3; 7.63–19.7).

**Discussion:**

The elevated rate of suicide after stroke persists beyond one year, highlighting the need for long-term screening for suicidality, especially in younger stroke survivors and those residing in low-income neighborhoods and with major depression after stroke.

## Background

Stroke survivors not only have physical disability but also experience major depression and other mental health disorders.^1^ However, mental health outcomes are often understudied when using population-based data.^1^ Stroke survivors have a 76% higher risk of suicide (composite of deliberate self-harm or death by suicide) compared to individuals without stroke, with a lower rate of suicide in cohort studies with longer duration of follow-up, highlighting the need for long-term follow-up when evaluating the suicide risk.^2^ The timing of suicide after stroke is also not well-known, making it challenging to develop interventions to prevent self-harm or suicide. Finally, sex differences in the rates of suicide after stroke are scarce,^3,4^ and the effects of other social determinants of health on the risk of suicide after stroke are seldom reported.^2,5^ More importantly, prior studies on suicide after stroke often do not exclude people with known depression or previous self-harm attempts.^2^

Rates of major depression among stroke survivors vary across studies based on different definitions of major depression, its ascertainment, or duration of follow-up,^6^ with many studies suggesting a higher rate of major depression in younger survivors^7^ and in female compared to male stroke survivors.^8^ However, most estimates are based on short durations of follow-up of up to 5 years after stroke, with little data on long-term risk.^9^ It is critical to study the role of major depression in the stroke–suicide association because it is common in those who self-harm, and it is a key risk factor for death by suicide.^10^

We aimed to compare rates of suicide in stroke survivors and the general population, identify the time of highest risk, and determine the sociodemographic factors associated with elevated suicide risk. We further assessed the modifying role of major depression on the association between stroke and suicide. Finally, we evaluated sex differences in these associations. We hypothesized that the risk of suicide would remain elevated beyond the first year after stroke, it would be more common in younger stroke survivors (below age 40 years) and females, and major depression would modify the association between stroke and suicide.

## Methods

### Setting

We used administrative health databases available at ICES (formerly known as the Institute for Clinical Evaluative Sciences), an independent, non-profit research institute in Toronto, Ontario. Multiple administrative health databases were linked using unique encoded identifiers and analyzed at ICES (details of the databases used in e-Table 1).

### Cohort creation

Ontario, Canada’s most populous province, has universal health insurance for all legal Ontario residents provided by the public third-party payer (i.e., the Ontario Ministry of Health); thus, all hospitalizations and emergency department visits are captured using health administrative databases. We identified all community-dwelling adult patients admitted to any acute care hospital in Ontario with the *most responsible or main* diagnosis of stroke using International Statistical Classification of Diseases and Related Health Problems [ICD]-10 codes for ischemic stroke (I63.x, I64, H34.1) or intracranial hemorrhage (I61.x) between January 1, 2008, and December 31, 2017. This definition has a sensitivity of 82.2%.^11^ We retained records of the first stroke for patients who had more than one stroke during the study period. Using a look-back window of 30 years, we excluded those with a history of stroke or transient ischemic attack,^11^ major depression,^12^ and history of deliberate self-harm^13^ using validated algorithms (supplemental e-Table 1).

We performed a 1:1 hard match between patients with stroke (cases) and controls identified from Ontario’s general population. Cases and controls were hard-matched based on age at the index date, sex, neighborhood-level income quintile, rural residence, and specific comorbidities defined according to validated algorithms: hypertension,^14^ diabetes,^15^ hyperlipidemia, atrial fibrillation,^16^ chronic obstructive pulmonary disease (COPD),^17^ and congestive heart failure (CHF).^18^ Doing so ensured that each stroke case was matched to a non-stroke control such that they both had the same variation in the covariates of interest.^19^ The index date among cases was the date of stroke hospitalization. For the index date in the controls without a stroke, first, we obtained a distribution of index dates in cases, and then we randomly assigned an index date to the controls from the distribution of index dates observed in the cases. This ensured that the cohort start dates for cases and controls are the same. Both cases and controls with a history of stroke or transient ischemic attack,^11^ major depression,^12^ and history of deliberate self-harm^13^ using validated algorithms (supplemental e-Table 1) were excluded.

### Outcome

Our primary outcome was suicide, defined as a composite of deliberate self-harm and death by suicide. Deliberate self-harm was defined as a hospitalization or emergency department visit with ICD-10 codes X60-X84, Y10-Y19, and Y28. Death by suicide was defined as deliberate self-harm leading to death or based on death certificates provided by the Office of the Registrar General Deaths database (ICD-10 codes X60-X84). This definition of suicide has been shown to have high specificity.^20^ We defined hospitalization for major depression as any hospitalization whose primary diagnosis was major depression using ICD-10 codes: F31.3-F31.5, F32.x, F33.x, and F34.1. This definition is precise (specificity ~90%).^21^ As this definition fails to identify cases of major depression not leading to hospitalization, in sensitivity analyses, major depression was defined as a hospitalization or ⩾2 ambulatory visits to a physician for major depression (using billing codes) in two consecutive years (sensitivity of 61.4%).^21^ Suicide and major depression were ascertained from the index date to December 31, 2018 (end of follow-up).

### Statistical analyses

We computed crude outcome rates (events/10,000 person-years) of major depression and suicide and the rate difference of major depression and suicide in cases vs controls during the duration of follow-up. We then reported the proportion of people who had major depression and suicide at 90 and 365 days after the index date. We constructed cumulative incidence curves (CIFs) for major depression and suicide and compared these between cases and matched controls using a CIF equality test.^22^ We accounted for the competing risk of death from any cause for the outcome of depression, and that of death from other causes other than by suicide for the outcome of suicide.

We then estimated several regression models. First, we used cause-specific hazard models to evaluate the association between stroke and suicide in the matched cohort. Given the matched design of our study, we did not adjust for any variables and used robust estimators when calculating confidence intervals to account for the matched nature of the sample.^23^ One key assumption of cause-specific models would be that the hazard of suicide associated with stroke remains constant over time. However, we hypothesized this not to be true. Thus, we conducted an analysis where we allowed the hazard ratio (HR) of suicide associated with stroke to vary as a function of time using restricted cubic splines to model the log-HR as a function of time.^24^ This analysis was to demonstrate how the hazard of suicide associated with stroke changes throughout follow-up and identify the time of highest risk of suicide.

Second, we added major depression as a time-varying covariate to these models to evaluate if adjusting for major depression attenuates the hazard of suicide in patients with stroke vs matched controls. We then evaluated the association between sociodemographic factors (age, sex, neighborhood income quintile, and rural residence) and the rate of suicide by restricting the cohort to cases only (stroke patients). We added hospitalization for major depression as a time-varying covariate in these models. To understand whether having major depression in the first 365 days after stroke was associated with higher rates of suicide compared to when it occurs after 365 days, we allowed the time-varying effects of major depression to vary so that we obtained the HR of suicide when major depression is diagnosed in the first 365 days following stroke vs after 365 days.

To evaluate the modifying effect of major depression on the stroke–suicide association, we added an interaction term between major depression (a time-varying variable) and stroke in the matched cohort. We repeated all the analyses stratified by sex to determine any sex differences. All analyses were carried out using SAS version 9.4.

### Standard protocol approvals, registrations, and patient consents

Datasets were linked deterministically using unique encoded identifiers and analyzed at ICES (formerly the Institute for Clinical Evaluative Sciences). The use of data in this project was authorized under section 45 of Ontario’s Personal Health Information Protection Act and did not require research ethics board approval.

### Data availability

Dataset from this study is held securely in coded form at ICES. While data sharing agreements prohibit ICES from making the dataset publicly available, access may be granted to those who meet the criteria for confidential access after an application process (www.ices.on.ca/DAS).

## Results

Of the 66,724 patients with stroke, 64,719 (97%) were successfully hard-matched 1:1 to the general population (e-Figure 1). The matching procedure led to cases and controls being identical on the specific characteristics used for matching (Table 1). Among the 64,719 pairs, 45.4% were female; the mean age was 71.4 years. The median duration of follow-up in the matched cohort was 2.3 years (Q1–Q3 = 0.9–4.5).

**Table 1.** Baseline characteristics of stroke patients matched to population-based controls.

Characteristics	Total	Stroke	Matched controls	Standardized difference
	N = 129,438	N = 64,719	N = 64,719	
Mean age in years (SD)	71.36 (13.67)	71.36 (13.67)	71.36 (13.67)	0
Female, n (%)	58,754 (45.4)	29,377 (45.4)	29,377 (45.4)	0
Neighborhood-level income quintile, n (%)				
Lowest (1st)	29,564 (22.8)	14,782 (22.8)	14,782 (22.8)	0
2nd	28,300 (21.9)	14,150 (21.9)	14,150 (21.9)	0
3rd	25,398 (19.6)	12,699 (19.6)	12,699 (19.6)	0
4th	23,766 (18.4)	11,883 (18.4)	11,883 (18.4)	0
Highest (5th)	22,410 (17.3)	11,205 (17.3)	11,205 (17.3)	0
Type of region, n (%)				
Large urban population ⩾ 100,000	100,432 (77.6)	50,216 (77.6)	50,216 (77.6)	0
Medium urban, population 10,000–100,000	12,652 (9.8)	6326 (9.8)	6326 (9.8%)	0
Rural, population < 10,000	16,354 (12.6)	8177 (12.6)	8177 (12.6)	0
Comorbidities, n (%)				
Hypertension	106,224 (82.1)	53,112 (82.1)	53,112 (82.1)	0
Diabetes	44,056 (34.0)	22,028 (34.0)	22,028 (34.0)	0
Atrial fibrillation	17,094 (13.2)	8547 (13.2)	8547 (13.2)	0
Dyslipidemia	15,366 (11.9)	7683 (11.9)	7683 (11.9)	0
COPD	12,556 (9.7)	6278 (9.7)	6278 (9.7)	0
CHF	18,004 (13.9)	9002 (13.9)	9002 (13.9)	0

SD, standard deviation; COPD, chronic obstructive pulmonary disease (COPD); CHF, congestive heart failure.

### Hospitalization for major depression after stroke

**Figure 1.**
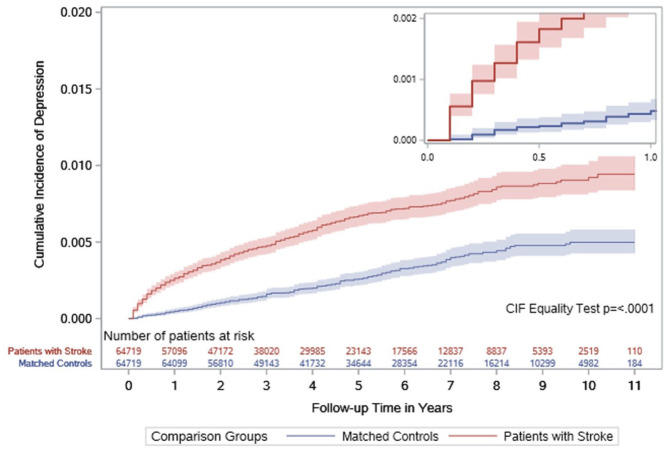
Cumulative incidence of hospitalization for major depression in patients with stroke and their matched controls. The smaller graph represents cumulative incidence of major depression in the first year.

The number of patients hospitalized for major depression during the 627,774 person-year follow-up period was higher in cases with stroke than in the matched controls (427 (0.7%) vs 197 (0.3%), P < 0001). The rate of hospitalization for major depression was higher in patients with stroke vs matched controls (crude rate 15.8 vs 5.5 per 10,000 person-years, rate difference 10.3; 95% CI (8.7–11.9), HR 2.76 (2.34–3.25)). The rate was high in the first year after the index date, but it remained elevated beyond the first year (Figure 1 and Table 2). In sex-stratified analyses, the rates of major depression remained higher in stroke cases than in matched controls, with smaller absolute and relative differences in females (crude rate 13.8 vs 5.4 per 10,000 person-years, rate difference 8.4 (6.2–10.6), HR 2.48 (1.91–3.23)) compared to males (crude rate 17.3 vs 5.6 per 10,000 person-years, rate difference 11.8 (9.6–14.0), HR 2.98 (2.39–3.72)).

**Table 2.** Incidence of major depression and suicide in the matched cohort.

Outcome of interest during follow-up	Stroke	Matched controls	P value
	N = 64,719	N = 64,719	
*Hospitalization for major depression, n (% of total population)*	*427 (0.7%)*	*197 (0.3)*	*<0.001*
⩽90 days	61 (14.3%)	6 (3.0%)	<0.001
⩽365 days^a^	166 (38.9%)	31 (15.7%)	<0.001
>365 days	261 (61.1%)	166 (84.3%)	<0.001
*Physician diagnosed major depression*^b^, *n (% of total population)*	*3460 (5.3%)*	*1879 (2.9%)*	*<0.001*
⩽90 days	905 (26.2%)	95 (5.1%)	*<0.001*
⩽365 days	1813 (52.4%)	350(18.6%)	*<0.001*
>365 days	1647 (47.6%)	1529 (81.4%)	0.03
*Suicide (deliberate self-harm or death by suicide), n (% of total population)*	*301 (0.5%)*	*135 (0.2%)*	*<0.001*
⩽90 days	36 (12.0%)	6 (4.4%)	<0.001
⩽365 days^a^	98 (32.6%)	25 (18.5%)	<0.001
>365 days	203 (67.4%)	110 (81.5%)	<0.001

a Event rate in ⩽365 days category includes events observed in the first 90 days.

b Includes hospitalizations, emergency room, and ambulatory visits with a diagnosis of major depression.

### Rates of suicide after stroke and the time of highest risk

**Figure 2.**
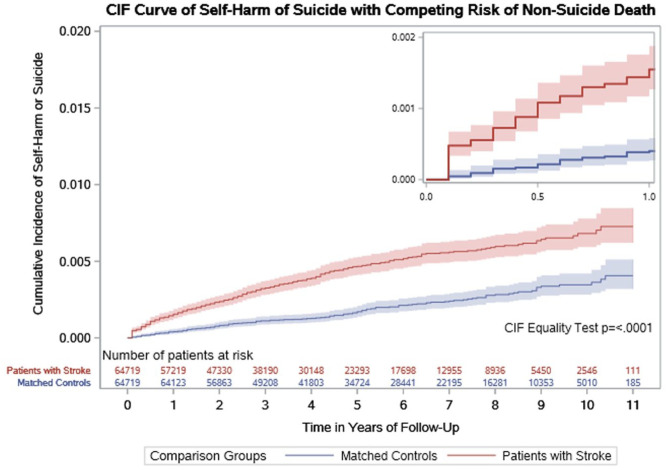
Cumulative incidence of suicide (deliberate self-harm or death by suicide) in patients with stroke and their matched controls. The smaller graph represents the cumulative incidence of suicide in the first year.

During 627,774 person-years follow-up, 436 (0.3%) cases and controls had an episode of suicide (404 had an episode of deliberate self-harm and 33 died by suicide). Suicide was more common in stroke survivors than matched controls (301 (0.5%) vs 135 (0.2%), P < 0.001), with only a third of the events (n = 98, 32.6%) occurring in the first 365 days in the stroke cohort (Table 2). Stroke was associated with a higher hazard of suicide than in matched controls (crude rate 11.1 vs 3.2/10,000 person-years, rate difference 7.9 (6.0–8.7) HR 2.87 (2.35–3.51)) [Figure 2]. Including major depression as a time-varying covariate in the regression analysis did not attenuate the higher hazard of suicide associated with stroke (HR 2.76; 2.26–3.37).

**Figure 3.**
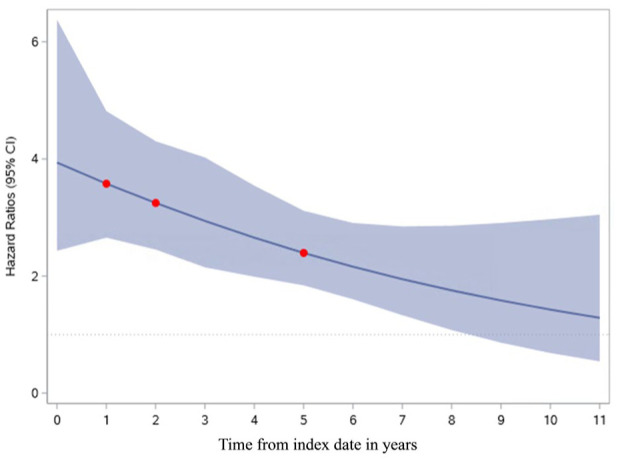
Changes in the hazard ratio of suicide in stroke survivors compared to matched controls during follow-up.

We found that the HR of suicide associated with stroke declined over time, albeit this decline was not linear (Figure 3). The HR of suicide was highest in the first year after the event (HR 3.94; 2.43–6.38), and it remained elevated well beyond the first year, becoming statistically non-significant after approximately 8 years from the index date (Figure 3).

In sex-stratified analyses, the rates of suicide were higher in stroke cases than in matched controls, with smaller absolute and relative differences in females (crude rate 9.6 vs 3.5 per 10,000 person-years, rate difference 6.2 (4.3–8.0), HR 2.70 (1.97–3.68)) than males (crude rate 12.2 vs 4.0 per 10,000 person-years, rate difference 8.2 (6.3–10.0), HR 2.98 (2.30–3.87)).

### Sociodemographic factors associated with suicide among stroke survivors

In multivariable models, after adjusting for major depression, suicide rates were higher in younger survivors compared to those older than 80 years (HR_18–40 vs_ _⩾_ _80 years_ 4.34 (2.48–7.61), HR_41–60_ 2.80 (1.93–4.07), and HR_61–80_ 1.47 (1.04–2.08)) (Table 3). Compared to those living in high-income neighborhoods, those in low-income neighborhoods (HR_lowest vs highest quintile_ 1.88 (1.30–2.70)) also had higher rates of suicide. Female sex (HR 0.87; 0.69–1.09) and rural residence were not associated with suicide (Table 3). While major depression was associated with a 12-fold increase in the rate of suicide (HR 12.26; 7.63–19.70), its effect did not vary substantially based on whether it was diagnosed in the first 365 days (HR 11.65; 5.96–22.73) vs after the first 365 days (HR 12.89; 6.75–24.61).

**Table 3.** Results of multivariable cause-specific hazard models evaluating the association between sociodemographic factors and depression (as a time-varying covariate) and the rate of suicide in stroke survivors (n = 64,719).

Characteristics of interest	Hazard ratio (95 % CI)
Age in groups (in years)	
18–40	4.34 (2.48–7.61)
41–60	2.80 (1.93–4.07)
61–80	1.47 (1.04–2.08)
⩾80 (reference)	1.00
Female vs male	0.87 (0.69–1.09)
Neighborhood-level income quintile	
1st	1.87 (1.30–2.70)
2nd	1.10 (0.73–1.65)
3rd	1.26 (0.84–1.88)
4^th^	1.12 (0.74–1.70)
5th (highest—reference)	1.00
Type of residence, number of people	
Small < 10,000	1.11 (0.81–1.53)
Medium urban ⩾ 10,000 and < 100,000	0.77 (0.51–1.16)
Large urban ⩾ 100,000 (reference)	1.00
Major depression	12.26 (7.63–19.69)
Comorbidities	
Hypertension	1.10 (0.83–1.46)
Diabetes	0.85 (0.66–1.09)
COPD	2.09 (1.52–2.88)
CHF	0.93 (0.64–1.36)
Atrial fibrillation	1.38 (0.97–1.97)
Dyslipidemia	0.94 (0.66–1.35)

COPD, chronic obstructive pulmonary disease; CHF, congestive heart failure.

### Effect modification by major depression

In the matched cohort, the association between stroke and suicide did not vary based on the presence or absence of major depression (P_stroke*depression_ = 0.51). This was true even when using a more sensitive definition of major depression (P_stroke*depression_ = 0.10). There was no modifying effect of major depression on suicide outcome in either sex (data not shown).

## Discussion

Using a population-based study of over 60,000 stroke survivors, we found a four-fold higher rate of suicide in stroke survivors compared to non-stroke controls in the first year, but the rate remained elevated beyond the first year. The overall two-fold higher rate of suicide associated with stroke remained elevated despite adjusting for major depression. Younger survivors, those living in low-income neighborhoods, and those who had a hospitalization for major depression were at a higher risk of suicide than others. The association between stroke and suicide did not vary by the presence of major depression, although the observed overall rate of suicide was low, suggesting that the interaction models were potentially underpowered.

Similar to other physical health conditions,^25^ the risk of suicide was high in the first year following stroke; however, despite a decline in the risk over time, it remained elevated for up to 8 years following stroke, suggesting a long-term impact of stroke on suicide risk. About two-thirds of cases of deliberate self-harm or death by suicide occurred after the first year following stroke. This is in contrast to previous work from Denmark, where the rate of suicide declined with time,^4^ but the estimates were derived based on a cohort between 1979 and 1993. Similarly, a study in Japan found a 10-fold increase in the risk of suicide after stroke, but the risk was only measured in the first 5 years after stroke.^26^ We found that the elevated rate of suicide after stroke was not only high in the first few years, but it persisted beyond 5 years. This is in keeping with a nationwide study in Denmark that found elevated risk of suicide after a neurological diagnosis beyond 5 years, but did not report stroke-specific risk from the time of diagnosis.^27^ Our findings call for ongoing screening and evaluation of suicidal ideation among stroke survivors.

As in previous work, we also found that younger stroke survivors had higher rates of suicide than older survivors.^4,5,26^ A few reasons for this may relate to differences between elderly and younger individuals: expectations of a disability-free life, access to tools or means for self-harm, and differences in psychiatric symptomatology.^28–31^ Rates of post-stroke depression are also higher in younger stroke survivors than in older survivors.^9^ However, it is important to note that, unlike previous work, our estimates were adjusted for major depression as a time-varying covariate. In line with previous work examining the general population^32,33^ and among stroke survivors,^5,34^ we also found a higher risk of suicide in stroke survivors residing in lower-income neighborhoods compared to those in higher-income neighborhoods, suggesting the role of income and neighborhood composition in elevated suicide risk.

Prior studies on sex differences in rates of suicide after stroke have shown mixed results. A study using the Swedish stroke registry found a higher incidence rate of suicide in male stroke survivors than their female counterparts,^5^ and another Swedish study using population-based data found that male stroke survivors were 19% more likely to attempt suicide than female stroke survivors, despite adjusting for sociodemographic factors.^5^ In contrast, suicide rates were similar in both sexes in studies undertaken in Denmark^4^ and Japan.^26^ We found slightly greater differences in the rates of suicide associated with having a stroke in males compared to females, but no association between sex and suicide among stroke survivors. Taken together, these findings highlight the need to further evaluate sex differences in suicide after stroke, using standardized outcome definitions across different jurisdictions.^6^

Depression after stroke is an important predictor of suicidal ideation or attempted suicide in stroke survivors based on previous observational studies.^5,35^ We confirm a higher rate of major depression in stroke survivors compared to matched controls, with this elevated risk persisting beyond the first year. We also demonstrate that major depression is associated with a 12-fold increased risk of suicide, regardless of whether it occurs within the first year after stroke or later. Yet, major depression did not modify the stroke–suicide association, and this was true even when using a more sensitive definition and for both sexes. In fact, up to 80% of those who had an episode of self-harm or died by suicide after a stroke did not have major depression, even when using a more sensitive definition. We propose a few possibilities for why we did not find a modifying effect of major depression. First, the overall suicide rate was very low in our cohort. Thus, it is possible that we did not have the statistical power to evaluate the interaction between stroke and major depression. Second, suicide in stroke survivors may not follow the pathway of major depression before suicide, which is found in other populations. In the general population, up to two-thirds of those who self-harm or engage in suicidal behavior have major depression, far exceeding the 20% observed in stroke survivors in our study.^36,37^ Second, diagnosing major depression in stroke survivors is challenging due to cognitive and language sequelae,^1^ underestimating the frequency and possibly the impact of post-stroke depression on the suicide risk. Imaging or biomarker studies to identify post-stroke depression may present one avenue to address these challenges.^38^ Finally, 13 (3.2%) of the 404 people who self-harmed in both cases and controls were diagnosed with major depression at the time of their presentation, suggesting that deliberate self-harm may be the first presentation of major depression in a small subset of people in our study, and thus, it is not possible to truly understand the role of major depression had it been diagnosed earlier.

Our study has several limitations. Suicide could only be reported as a composite outcome due to small numbers. Thus, differences in the association between sociodemographic factors and deliberate self-harm and death by suicide could not be ascertained. Furthermore, owing to an overall low rate of suicide, we could not evaluate the interaction between sociodemographic characteristics and their impact on suicide. Our definition of major depression cannot identify patients with depressive symptoms who are managed by nurse practitioners, psychologists, or social workers, and those who were on antidepressants or other psychiatric drugs. When using a sensitive definition, we are using physician billing codes, which are known to have misclassification bias and often include other diagnoses such as anxiety. We selected matching healthy control populations based on sociodemographic characteristics and prevalent comorbidities, which may lead to biased estimates if the distribution of other life-altering conditions associated with suicide (e.g., myocardial infarction or sepsis) is lower. We also could not account for social factors such as social support, loneliness, or social isolation, nor could we account for stroke-specific factors such as location and severity of stroke, or functional disability after stroke, potentially leading to residual confounding and limiting our ability to infer causality.^1,31^ Finally, the lack of statistical significance in the interaction term between stroke and major depression should not be interpreted as the absence of a modifying effect. This may reflect the small number of events in our cohort, despite long-term follow-up and use of a population-based sample.

In summary, the higher hazard of suicide after stroke is sustained beyond the first year after stroke, especially in younger stroke survivors and those living in low-income neighborhoods. Ongoing screening beyond the first year for depressive symptoms and suicidal ideation may be necessary to reduce the burden of suicide in stroke survivors.

## Supplemental Material

sj-docx-1-wso-10.1177_17474930251379165 – Supplemental material for Increased risk of suicide after stroke: A population-based matched cohort study Supplemental material, sj-docx-1-wso-10.1177_17474930251379165 for Increased risk of suicide after stroke: A population-based matched cohort study by Manav V Vyas, Claire de Oliveira, Gustavo Saposnik, Peter C Austin, Amy YX Yu, Olivia Haldenby, Jiming Fang, Corinne E Fischer, David Lipson, Fatima Quraishi, Moira K Kapral and Venkat Bhat in International Journal of Stroke
