# Supplementary material for: Increased risk of suicide after stroke: A population-based matched cohort study
Source: Int J Stroke. 2025 Sep 5;21(4):485–94. doi: 10.1177/17474930251379165 (PMC13009222; doi:10.1177/17474930251379165)
Supplement: sj-docx-1-wso-10.1177_17474930251379165 – Supplemental material for Increased risk of suicide after stroke: A population-based matched cohort study [file sj-docx-1-wso-10.1177_17474930251379165.docx]

**e-table 1**. Data sources and operationalization of covariates

| **Variable** | **Type** | **Definition** | **Data source** |
| --- | --- | --- | --- |
| Incident stroke (ischemic or intracerebral hemorrhage) | Binary | Hospitalization or emergency department with a corresponding ICD-10 code in the main diagnostic field: H34.1, I63, I64, I61 | CIHI-DAD or NACRS |
| **Outcome definitions** |  |  |  |
| Hospitalization for major depression |  | *DAD/NACRS*  ICD-9 codes: “296.2x, 296.3x, 296.5x, 296.82” [flag these], 309, and 311  *OMHRS*  < 2017: ICD 9 code: 311.x Provisional = 6  2017-2019: ICD9 code: Depressive [296.2x, 296.3x, 296.9x, 300.4x, 311.x, 625.4x. provisional=4  >2019: ICD-10 codes: Depressive [F32, F33, F34.81, F34.1, F06.31, F06.32], Provisional = 3, 4 | CIHI-DAD or NACRS or OMHRS |
| Sensitive definition of major depression |  | 2 depression claims in a two-year period (by any physician)  OHIP billing codes: 309 (adjustment) and 311 (depression)  +  above |  |
| Deliberate self-harm |  | Hospitalization or emergency room visit with any diagnostic code: ICD-9 codes: E950-959 or ICD-10 code: X60-X84, or Y28 or Y10-Y19. |  |
| Death by suicide |  | < 2013: (ICD-10 code X60–X84).  ≥ 2013: manner_of_death = 4 | ORGD |
| Death | Binary | Along with date of death | RPBD |
| **Covariates** |  |  |  |
| Age | Continuous | Based on date of birth of participants | RPDB |
| Sex | Binary | Female or male, male used as the comparison group | RPDB |
| Neighbourhood-level income | Quintiles | Obtained by linking census information from 2006 and 2011 to postal-code files | PCCF and Census |
| Region of residence | Categorical | dissemination areas with population of less than 10,000 people as “rural residents”, large urban = > 100,000, and medium urban = >10,000 and < 100,000 | PCCF and Census |
| Hypertension | Binary | ≥ 1 Hospitalization for hypertension **OR**  ≥ 2 physician claims in a two-year period **OR**  1 physician claim followed by another physician claim or hospitalization within two years. | Multiple sources |
| Diabetes | Binary | ≥ 3 physician diagnostic code (250) in a one-year period | Multiple sources |
| Chronic obstructive pulmonary disease (COPD) | Binary | ≥1 Hospitalization for COPD **OR**  ≥ 3 physician claims in a two-year period | Multiple sources |
| Congestive heart failure (CHF) | Binary | ≥ 1 Hospitalization **OR**  1 physician claim in emergency visit or outpatient clinic, followed by ≥ 1 Hospitalization, ER visit, or physician claim within one year. | Multiple sources |
| Dyslipidemia | Binary | Based on OLIS database:  Levels above the threshold (2.5 mg/dL) will be considered to have hyperlipidemia **OR**  ODB claims for one of the statins (using DIN list) | Multiple sources |
| Atrial fibrillation | Binary | 1 hospitalization (CIHI-DAD) or 1 emergency room visit (NACRS/SDS), ICD-10 (2002 onwards) – I48; ICD-9 (pre-2002) – 427.31 or 427.32 **OR**  cardioversion (without physician billing codes) – using billing code Z437 | Multiple sources |
|  |  |  |  |

**Abbreviations**: OHIP – Ontario Health Insurance Plan – physician claims database, CIHI-DAD – Canadian Institute for Health Information- Discharge Abstract Database, RPDB – Registered Persons Database, PCCF – postal code conversion files, NACRS – National Ambulatory Care Reporting System, ORGD – Office of the Registrar General Deaths database, OMHRS – Ontario Mental Health Reporting System

**e-figure 1.** Flow Diagram of Patient and Matching

Ontario residents aged 18 to 105 years with incident stoke between Jan 1, 2008 and Dec 31, 2017

N = 137,599

Keeping only first stroke event during ascertainment window

N = 120,926

Keeping those who live in the community and not in a long-term care home

N = 113,721

Keeping those with first-ever events

N = 90,649

Keeping those without history of depression

N = 66,533

Excluding second events

n = 16,673

Excluding those residing in long-term care homes, n = 7205

Excluding those with history of TIA or stroke, n = 23,072

Excluding those with history of depression, n = 24,116

Keeping those without history of deliberate self-harm

**N = 66,274 [sample pre-match]**

Excluding those with history of self-harm, n = 259

Final sample of patients with stroke matched to general population

N = 64,719

**Matching** 1:1 to Ontario’s population without history of stroke, TIA, depression and self-harm
